# Management in robot-assisted radical prostatectomy patients with recto-urethral fistulas: the York–Mason technique

**DOI:** 10.1007/s00345-025-05996-5

**Published:** 2025-10-11

**Authors:** Sophia H. van der Graaf, Esther M. K. Wit, Geerard L. Beets, Brechtje A. Grotenhuis, Ton A. Roeleveld, Jakko A. Nieuwenhuijzen, André N. Vis, Pim J. van Leeuwen, Henk G. van der Poel

**Affiliations:** 1 Department of Urology, Netherlands Cancer Institute—Antoni van Leeuwenhoek Hospital (NCI-AVL), Amsterdam, the Netherlands; 2 Department of Urology, Amsterdam University Medical Centers Location VUmc, De Boelelaan 1117, 1081 HV Amsterdam, the Netherlands; 3 Prostate Cancer Network Netherlands, Amsterdam, the Netherlands; 4 Department of Surgery, Netherlands Cancer Institute—Antoni van Leeuwenhoek Hospital (NCI-AVL), Amsterdam, the Netherlands; 5 Department of Surgery, Maastricht University Medical Centre, Maastricht, the Netherlands; 6 GROW Research Institute for Oncology and Reproduction, Maastricht University, Maastricht, the Netherlands; 7 Department of Urology, Noordwest Ziekenhuisgroep, Alkmaar, the Netherlands

**Keywords:** Prostate cancer, Robot-assisted radical prostatectomy, Recto-urethral fistulas, York–Mason technique, Complications

## Abstract

**Purpose:**

To present the outcomes of the York–Mason procedure for managing recto-urethral fistula (RUF) after robot-assisted radical prostatectomy (RARP).

**Methods:**

A single-center retrospective cohort study was conducted at the Antoni van Leeuwenhoek Hospital–Netherlands Cancer Institute. Between January 2011 and May 2024, 12 cases of RUF following RARP, all treated with the York–Mason technique, were identified. Initial treatment involved conservative management with prolonged catheterization. If unsuccessful, surgical intervention followed. The York–Mason procedure is a posterior transsphincteric approach, providing direct access to the fistula through healthy tissue, with precise layer-by-layer closure of the sphincter complex. Successful repair was defined as the absence of anal urinary loss, the absence of fecal material in the catheter bag or pneumaturia, and the absence of leakage on cystogram.

**Results:**

Of the 12 cases of RUF, 10 occurred in patients who underwent RARP without radiotherapy (RARP-only), and 8 of these underwent York–Mason procedure as initial surgical treatment, achieving a 100% success rate after a median follow-up of 5.1 years (SD 2.9). The remaining two RARP-only patients initially underwent other surgical interventions, after which one achieved successful closure with subsequent York–Mason repair. The other two patients received both RARP and radiotherapy before fistula detection– one received radiotherapy prior to RARP, and one after. York–Mason was effective when radiotherapy preceded RARP.

**Conclusion:**

The York–Mason procedure provides promising results for managing RUFs in patients who have undergone RARP without salvage radiotherapy. Its effectiveness is particularly evident when used as the first surgical intervention.

## Introduction

Robot-assisted radical prostatectomy (RARP) is a widely used treatment for men with clinically localized prostate cancer (PCa) [1, 2]. This technique is generally safe and, aside from the risk of decreased functional outcomes such as urinary incontinence and erectile dysfunction, associated with a relatively low risk of complications [3]. The complications that do occur vary, such as urine leakage, with most being of low grade [4, 5]. However, a particularly rare complication with a high impact on loss of quality of life is recto-urethral fistula (RUF) [6].

The incidence of RUF following primary RARP is low, generally reported to be up to 0.53% [7]. In contrast, after salvage prostatectomy, reported rates are ranging from 2% to 16% [8, 9]. RUF is an opening between the rectum and the bladder or the urethra, typically presents with fecaluria, pneumaturia, and urinary drainage per anus, often confirmed by leakage on a cystogram [10, 11]. Approximately 60% of cases are caused by iatrogenic lesions occurring during surgical procedures, radiation therapy, brachytherapy, and cryotherapy [10]. RUF can appear in patients after RARP, mainly due to unidentified rectal injuries during surgery, or secondary to an identified rectal injury sutured intraoperatively [11]. These fistulas typically manifest a few days or a few weeks postoperatively, although the time frame can vary widely [12]. The RUFs secondary to radiation therapy appear later, and may be delayed by months or years after treatment [11]. The presence of RUF increases morbidity rates, prolongs hospital stay, escalates health care costs and impairs patients’ quality of life [13].

For RUFs following RARP without a history of radiotherapy, treatment options include both conservative management and surgical approaches. In contrast, RUF secondary to radiation therapy rarely close spontaneously, warranting immediate surgical intervention. Conservative management typically involves urethral catheterization aiming for spontaneous closure of the fistula [14]. If fecal leakage persists after three months and there is no endoscopic improvement, surgical intervention becomes necessary [11, 14, 15]. Although various surgical approaches, including perineal, transrectal, transsphincteric, transanorectal and combined abdomino-perineal repairs, have been described, no standardized approach exists due to the rarity of RUF. Previous studies have shown that patients who received radiotherapy are at increased risk of developing fistulas and may respond differently to treatment, categorizing them as a distinct subgroup [2, 11, 16, 17]. Additionally, patients with fistulas resulting from salvage cryotherapy or HIFU represent another unique group. These fistulas are generally unsuitable for surgical fistula repair, placing them beyond the scope of this article [11].

In this paper, we present our 13-year experience with the York–Mason technique for surgical management of RUF in RARP patients, highlighting outcomes and specific considerations. The York–Mason is a posterior transsphincteric approach that provides direct access to the fistula through healthy tissue, with precise layer-by-layer closure of the sphincter complex.

## Methods

A single-center retrospective cohort study was conducted at the Antoni van Leeuwenhoek Hospital—Netherlands Cancer Institute (AVL-NCI). The study was approved by the institutional review board of the AVL-NCI (IRBd19-248) and conducted in accordance with the ethical standards of the 1964 Declaration of Helsinki [18]. The need for informed consent was waived.

### Study population

We retrospectively analyzed PCa patients who underwent a York–Mason for RUF following RARP at AVL–NCI between January 2011 and May 2024. Patients of all PSA values and tumor stages were included.

### Data

The following variables were collected for each patient: age (years), clinical tumor stage (cT-stage), radiological tumor stage (mT-stage), Gleason score in biopsies, history of radiotherapy (prior to or following RARP), pelvic lymph node dissection (PLND), noted surgical comments, pathological tumor stage (pT-stage), pathological nodal stage (pN-stage), R0 status, date of RUF diagnosis, diagnostic method for RUF, treatment for RUF, date of York–Mason procedure, noted comments about the York–Mason procedure, surgeon performing the York–Mason procedure, and reported functional outcomes after the York–Mason procedure. Bowel function after colostomy reversal was assessed using the Low Anterior Resection Syndrome (LARS) score. The LARS score consists of five questions evaluating aspects of bowel function: control of flatus, incontinence for liquid stools, frequency of bowel opening, clustering of stools, and urgency. The total score ranges from 0 to 42, with higher scores indicating more severe symptoms of bowel dysfunction. Scores are categorized as follows: 0–20 indicates no LARS, 21–29 minor LARS, and 30–42 major LARS [19].

### Diagnosis of RUF

**Fig. 1:**
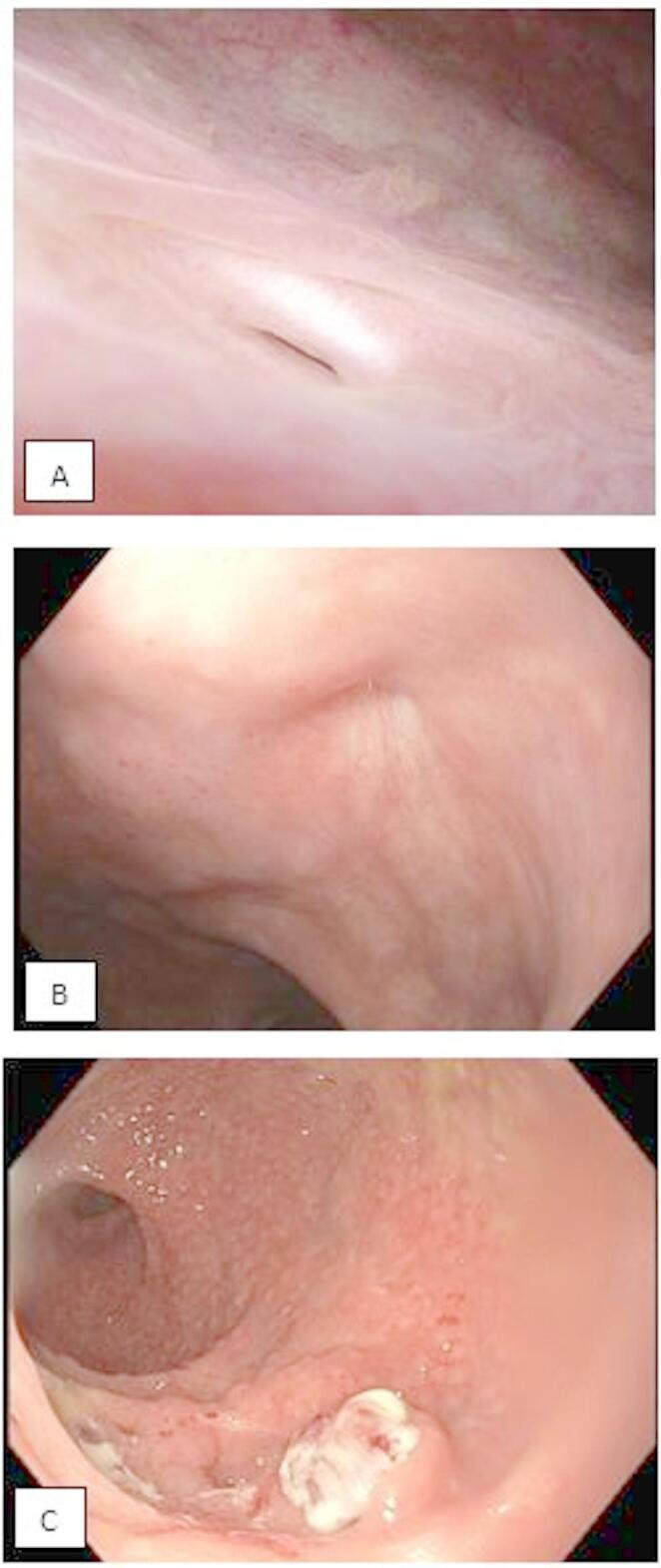
Three images with findings from one patient taken during the trajectory for RUF, including both the diagnostic and post-treatment phases. **A** Cystoscopy image during the diagnostic trajectory: fistula opening, estimated to be 2 mm, appears to be epithelialized and surrounded by healthy tissue. **B** Proctoscopy image during the diagnostic trajectory: fistula opening, estimated to be 2 mm, approximately 3 cm from the anal ring. **C** Proctoscopy image after a successful York–Mason procedure: a small scar with granulation tissue anteriorly, 2 cm above the sphincter.

The clinical presentations were complaints of fecaluria and/or pneumaturia and watery stool prompts further investigation. Initially, the distance of the fistula from the anal ring was assessed through rectal examination by palpating an indentation. In more recent cases, surgeons directly performed a proctoscopy and cystoscopy to confirm the presence of the fistula (Fig. 1A, B).

### Management of RUF

Prior to surgical intervention, the initial approach at AVL–NCI was conservative management. The goal of this non-surgical strategy was to promote spontaneous fistula closure, primarily through prolonged transurethral catheterization with a Foley catheter, typically maintained for at least three months [11, 15]. During this period, patients were monitored through regular hospital visits for catheter changes, complemented by telephone consultations to track symptoms. After three months, a repeat cystoscopy and cystogram were performed to evaluate whether the fistula had closed or persisted. If epithelialization was observed on cystoscopy, spontaneous closure was deemed unlikely. Surgical intervention was pursued when conservative management failed, as defined by persistent fistula-related symptoms and confirmation of the fistula via endoscopic evaluation.

**Fig. 2:**
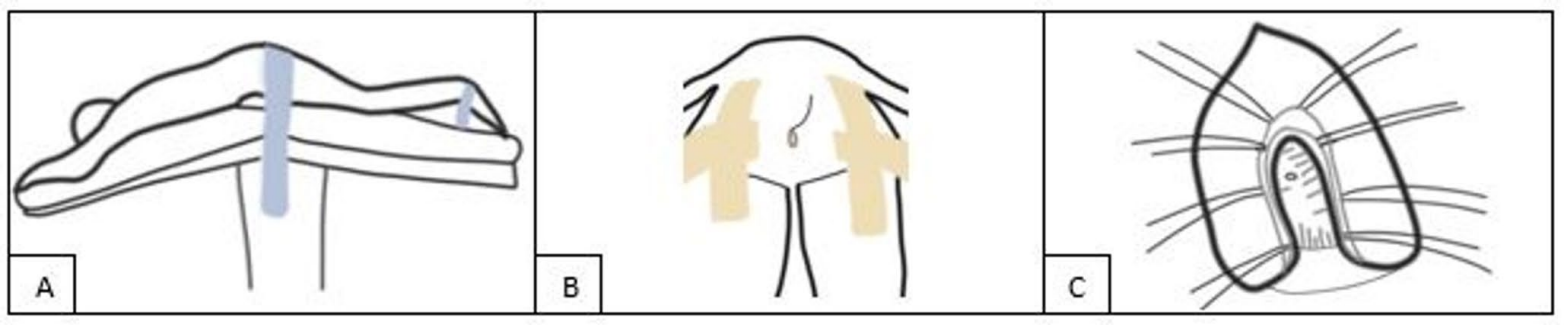
Three schematic illustrations of the position and incision during the York–Mason procedure. **A** Schematic illustration showing the prone patient position during the York–Mason procedure with fixation straps (blue). **B** Schematic illustration of the incision site (gray), extending from the anus onward. Both gluteal regions are held back with adhesive tape (skin-colored). **C** Schematic illustration showing the rectal wall, puborectal muscle, internal and external sphincters with different muscle layers, with multiple sutures to identify the fistula.

All patients received a diverting colostomy, either prior to or during the same session as the fistula repair. Colostomy reversal was considered after a minimum interval of three months, once successful RUF repair had been confirmed. The York–Mason procedure has been described previously [20]. In our institution the procedure is performed using a right-sided, U-shaped incision rather than a midline approach. This modification provides optimal exposure of the fistula site. The other steps of the procedure are as follows: the patient was placed in a prone position, with the hips flexed at the level of the femoral thigh joint (Fig. 2A). Both gluteals were retracted using adhesive tape and a right-sided incision was made adjacent to the coccyx, extending transversely through all layers of the anal sphincter (Fig. 2B). Each layer was marked with sutures and tagged separately with clamps and gauze for later identification (Fig. 2C). The fistula was then identified anteriorly, and hydro dissection using a lidocaine adrenaline solution was performed to facilitate tissue separation. The fistula tract was excised, and the mucosa was mobilized from the underlying tissue. The bladder was closed using sutures, followed by a leak test with sterile water. The rectal mucosal layer was closed over the fistula. The wound was closed in layers, using the previously marked sutures as guides to ensure proper reapproximation of the corresponding tissue layers. Finally, the subcutaneous tissue was closed, followed by intracutaneous closure of the skin.

Following the York–Mason, a Foley catheter was inserted in all patients, and three weeks postoperatively a cystogram was performed to evaluate the success of the repair and confirm the absence of any leakage, with confirmation in some cases by proctoscopy (Fig. 1C).

### Definition of successful fistula repair

In our institution, successful fistula repair is defined as the absence of anal urinary loss, the absence of fecal material in the catheter bag or pneumaturia, and the absence of leakage on cystogram performed 3 weeks after surgery. Success is confirmed by the absence of these symptoms from 3 months postoperatively onwards. Reversal of fecal diversion was considered at least 3 months after definitive successful RUF repair, provided that the patient is continent for feces.

### Statistical analysis

Continuous variables were reported as medians with interquartile ranges (IQR). Nominal variables were reported as frequencies and percentages (%). Statistical analysis was performed using SPSS version 29.0 (IBM).

## Results

### Baseline characteristics

A total of 12 patients who underwent RARP and developed RUF between January 2011 and May 2024 were included in this study. Baseline characteristics are summarized in Table 1. The mean age of the cohort was 67 years (SD 4.6), and the mean BMI was 26.2 (SD 3.5). The majority of patients (66%) had either a cT1c or cT2 stage, with an even distribution between both; one patient was classified as cT3. Pathological stages were more varied: 33% were pT2, 25% pT3a, and 25% pT3b, with two patients having unknown pathological stage. Additionally, 50% of patients had a PSM following RARP. The median follow-up duration for the cohort was 7.0 years (IQR 3.0–8.0).

**Table 1:** Baseline characteristics

	RARP patients with RUF treated by York–Mason (*n* = 12)
*Age (years)*, mean (SD)	67 (4.6)
*BMI (kg/m*^*2*^*)*, mean (SD)	26.2 (3.5) Missing *n* = 5 (24)
*Died during FU*, *n* (%)	3 (25)
*cT-stage*, *n* (%)	
T1c	4 (33)
T2	4 (33)
T3	1 (8)
Unknown	3 (25)
*pT-stage*, *n* (%)	
T2	4 (33)
T3a	3 (25)
T3b	3 (25)
Unknown	2 (16)
*Resection status*, *n* (%)	
R0 resection	2 (16)
R1 resection	6 (50)
Unknown	4 (33)
*Initial treatment PCa*, *n* (%)	
Prostatectomy	9 (75)
Prostatectomy + PLND	2 (16)
Radiotherapy	1 (8)
*Colostomy before YM*, *n* (%)	12 (100)
*Interval RUF diagnosis and YM (months)*, mean (SD)	13.2 (14.8)
*Follow-up (years)*	
Mean (SD)	6.3 (3.6)
Mediaan (IQR)	7.0 (3.0–8.0)

Some percentages may not sum to 100 because of rounding

*RUF* recto-urethral fistula, *SD* standard deviation, *BMI* body mass index, *cT-stage* clinical tumor stage, *pT-stage* pathology tumor stage, *R0 resection* radical resection, *R1 resection* irradical resection, *PLND* pelvic lymph node dissection, *RARP* robot-assisted radical prostatectomy, *YM* York–Mason, *IQR* interquartile range

**Fig. 3:**
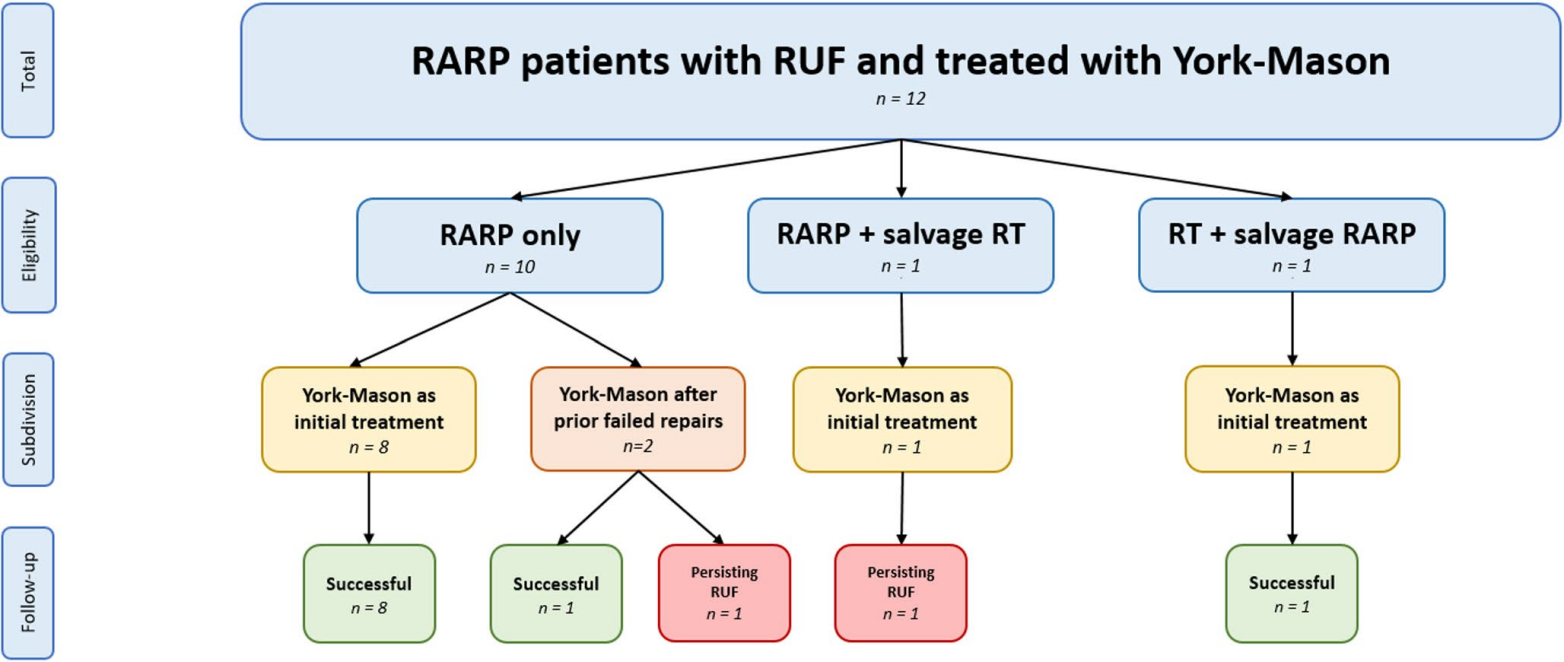
Flowchart RUF patients. *RARP* Robot-assisted radical prostatectomy, *RT* radiotherapy, *RUF* recto-urethral fistula

The cohort of 12 RUF patients was initially divided into three groups: 10 patients treated with RARP-only and two patients received both radiotherapy and RARP. The latter group was further subdivided into one patient who underwent radiotherapy prior to RARP and one who underwent radiotherapy after RARP (Fig. 3).

### RARP-only patients

Among the 10 RARP-only patients, intraoperative rectal injury was identified in four cases and primarily closed with sutures. Unfortunately, all four developed a RUF, which was detected within the first postoperative week. In the remaining 6 patients, RUF was diagnosed at a mean interval of 8.3 weeks after surgery (SD 8.3).

Eight patients underwent the York–Mason procedure as their initial surgical fistula treatment, with a median interval of 6.6 months (SD 2.1) from RUF diagnosis. In three of these patients, colostomy reversal was not performed due to patient preference, ulcerative colitis, or death from unrelated comorbidities. In the remaining five patients, colostomy reversal was achieved at a mean interval of 6.3 months (SD 5.0) following the York–Mason procedure. No urinary loss per anus or fecal incontinence was reported, corresponding to a 100% success rate in this subgroup over a median follow-up of 5.1 years (SD 2.9). Post-reversal bowel function, assessed using the LARS score, indicated low symptom burden in all five patients (score range: 0–20).

The other two patients in the RARP-only group received alternative initial fistula treatments. The first patient underwent an abdominoperineal repair, but experienced recurrence and subsequently underwent a York–Mason procedure. This procedure was also unsuccessful, and the patient ultimately required total pelvic exenteration with Bricker diversion and definitive colostomy. The second patient in this group initially underwent neo-anastomotic urethral reconstruction combined with perineal fistula repair after RARP, which was unsuccessful. A definitive colostomy was then created. Due to persistent fistula-related symptoms and urinary incontinence, the patient subsequently underwent cystectomy with Bricker diversion, during which fistula closure was not feasible. Eventually, a York–Mason repair was performed, which successfully closed the fistula.

### RARP + salvage radiotherapy patient

This group consisted of one patient, who received salvage radiotherapy after RARP for recurrent PCa. This patient was diagnosed with RUF following salvage radiotherapy, which was identified as the cause of the fistula. He underwent a York–Mason procedure, which was unsuccessful and required multiple additional surgeries.

### Radiotherapy + salvage RARP patient

This group consisted of one patient, who underwent salvage RARP one year after primary brachytherapy. Seventeen months after RARP, a York–Mason was performed as the initial surgical fistula treatment, after a colostomy had already been created. The repair was successful, and the colostomy was reversed within one year. This patient scored low on the LARS score (0–20 points).

## Discussion

This retrospective cohort study evaluated RUF cases in RARP patients from January 2011 to May 2024. Twelve cases of RUF were identified, all treated with the York Mason procedure. Ten occurred in patients treated with RARP only, and 8 of these underwent the York–Mason procedure as initial surgical treatment, achieving a 100% success rate. In contrast, its efficacy was limited in patients who had undergone radiotherapy after RARP, as well as in those for whom York–Mason was not the initial surgical intervention, highlighting the negative impact of salvage radiotherapy and previous failed surgeries on tissue healing and surgical outcomes.

The incidence of RUF in our cohort was 0.3% (12 out of 3,693 RARP procedures), including predominantly primary RARP cases with only one case after salvage RARP. This aligns with reported rates following primary RARP (up to 0.53%) and remains notably lower than those reported following salvage prostatectomy (2%–16%) [7–9]. In our study, four rectal injuries were identified intraoperatively and primarily closed, yet al.l of these patients developed RUF postoperatively. However, the majority of RUFs in our cohort occurred without intraoperative recognition of a rectal injury. This pattern is consistent with existing literature, where most RUFs are attributed to unrecognized rectal injuries during RARP [21]. These findings emphasize the importance of optimizing intraoperative techniques to minimize the risk of rectal injury and to enhance early recognition when it occurs [22]. In addition, when a rectal injury is identified, clear and immediate management appears critical, as this is considered the most determining factor in preventing RUF formation [23]. Some studies advocate for immediate drainage and interruption of surgery, while others suggest completing RARP with additional measures such as colostomy [24, 25]. The absence of systematic perioperative documentation of rectal injuries at our institution limits further analysis of this aspect.

Accurate diagnosis plays a critical role in achieving successful outcomes in RUF treatment. In our cohort, both cystoscopy and cystogram were performed and provided invaluable for diagnosing and localizing fistulas, consistent with findings in the literature [26–28]. The addition of proctoscopy in recent years has further enhanced surgical preparation by providing detailed insights into fistula size and location. These diagnostic improvements likely contributed to better selection of RUF patients for the most suitable repair strategies, thereby potentially improving surgical outcomes.

The York–Mason procedure demonstrated promising outcomes in our cohort when applied as a primary intervention in non-irradiated patients. Our success rate (100%) is comparable to previous reports using the York–Mason: one study reported by Dal Moro et al. and another by Crippa et al. each described 100% success in seven patients undergoing York–Mason. A larger series by Hadley et al. showed a success rate of 93%, while Bergerat et al. reported 80% success. In all cases, failures were associated with prior radiotherapy or when York–Mason was not used as the initial surgical approach. Collectively, these studies reinforce the value of early, definitive intervention in appropriately selected patients.

Management of RUF becomes considerably more complex in patients who have received radiotherapy. Radiotherapy significantly alters tissue properties, including reduced vascularization and increased fibrosis, which can impair healing and complicate surgical interventions [2, 11, 16, 17]. These histopathological changes can severely limit the success of conventional repair techniques such as the York–Mason. In our cohort, the single case of RUF following post-RARP radiotherapy was likely attributable to radiation-induced damage rather than the surgical procedure itself [2]. Given these challenges, conventional techniques like York–Mason may be insufficient. Based on our experience, we advocate for more aggressive approaches in irradiated patients, such as total pelvic exenteration with ORAM reconstruction.

Another aspect of RUF management is the use of a diverting colostomy during the York–Mason procedure. In our cohort, all patients underwent diverting colostomy, either prior to or during the York-Mason procedure. This approach aims to minimize fecal contamination and support optimal wound healing. However, there is still debate on it in the literature. Some authors argue that in selected cases, particularly with small fistulae (< 2 cm) or in patients with the RUF diagnosis 6–8 weeks postoperatively, primary repair without colostomy may be effective [28, 29]. In cases without a diverting colostomy, the patient is then placed on no oral intake until bowel function returns. In cases of large fistulous tract, or with previous radiotherapy, the creation of fecal diverson with colostomy is more advised [26]. These contrasting approaches suggest that while fecal diversion may not be universally required, it remains a prudent strategy in high-risk patients. Further research is needed to better define selection criteria for omitting colostomy in RUF management.

In our institute, the initial management of RUF following RARP consists of conservative treatment, involving prolonged bladder catheterization. Although existing studies are limited by small sample sizes, several have reported promising outcomes with this approach. Popov et al. described spontaneous closure in four out of five patients (80%) treated conservatively with catheterization, dietary restrictions, antibiotics and bowel rest [30]. Similarly, Noldus et al. reported complete healing in 7 out of 12 patients managed with prolonged catheter drainage alone, without surgical intervention [31]. These findings suggest that, in selected cases, conservative treatment may offer a viable, non-invasive alternative prior to surgical repair.

Although the York–Mason approach remains widely utilized, several alternative techniques have been described. These include transperineal repairs with interposition flaps, gracilis muscle transposition, and transanal minimally invasive surgery (TAMIS), each offering specific advantages and limitations. According to a comprehensive review by Chen et al., the choice of surgical strategy should be tailored based on factors such as fistula size and location, prior radiation therapy, and surgeon experience [21]. While no single technique has proven universally superior, literature suggests that flap-based approaches may offer higher success rates in irradiated patients, whereas TAMIS might reduce morbidity in select non-irradiated cases. Future comparative studies are warranted to better define optimal surgical pathways across diverse patient populations.

This study has several limitations. A key limitation is the small sample size of the cohort (12 patients), with only two patients with prior radiotherapy, which restricts the generalizability of subgroup analyses. Due to the retrospective design, there is a risk of selection and information bias, limiting the external validity of the findings. Another limitation is the absence of a standardized protocol for managing RUF, leading to variability in treatment approaches. Furthermore, as our center has focused primarily on the York–Mason procedure due to the rarity of recto-urethral fistulas, we could not perform a direct comparison with alternative surgical techniques. This limits the ability to evaluate the relative merits of different approaches. Future multicenter studies could help address this gap by comparing various surgical strategies. Additionally, the multicenter nature of our network resulted in incomplete follow-up data, potentially affecting the accuracy of our outcomes. Lastly, the limited success of the York–Mason procedure after radiotherapy warrants further investigation. Future studies need to explore innovative approaches to improve outcomes in this challenging subgroup. In this context, the emergence of minimally invasive surgical techniques, such as corrections using a TAMIS port, may offer less invasive alternatives [32, 33]. As these techniques evolve, the York–Mason procedure may increasingly be considered too invasive in selected cases. Moreover, emerging technologies like single-port Da Vinci robotic surgery and transvesical approaches may hold promise for less invasive fistula repair, although evidence is still lacking.

### Conclusion

The York–Mason procedure provides promising results for managing RUFs in patients who have undergone RARP without salvage radiotherapy, where the fistula is attributed to surgical injury. Its effectiveness is particularly evident when used as the first surgical intervention.

## Data Availability

The data that support the findings of this study are available from the corresponding author upon reasonable request.
